# Revealing the charge carrier kinetics in perovskite solar cells affected by mesoscopic structures and defect states from simple transient photovoltage measurements

**DOI:** 10.1038/s41598-020-74603-x

**Published:** 2020-11-05

**Authors:** Rahmat Hidayat, Adhita Asma Nurunnizar, Alvin Fariz, Herman, Erlyta Septa Rosa, Shobih, Tomohisa Oizumi, Akihiko Fujii, Masanori Ozaki

**Affiliations:** 1 Institut Teknologi Bandung, Physics of Magnetism and Photonics Research Division, Physics Program Study, Faculty of Mathematics and Natural Sciences, Jl. Ganesha 10, Bandung, West Java 40132 Indonesia; 2 Research Center for Electronics and Telecommunication, Indonesian Institute of Sciences, Jl. Sangkuriang Komp. LIPI Gd.20, Bandung, West Java 40135 Indonesia; 3 Department of Electrical, Electronics, and Information Engineering, Faculty of Engineering, Osaka University, Yamada-Oka 2-1, Suita, Osaka 565-0871 Japan

**Keywords:** Materials science, Nanoscience and technology, Optics and photonics, Energy science and technology, Energy harvesting, Devices for energy harvesting, Physics, Electronics, photonics and device physics

## Abstract

This report shows that, by using simple transient photovoltage (TPV) measurements, we can reveal a significant correlation between the TPV decay characteristics and the performance of these perovskite solar cells. TPV decay seems to be composed of a rising part in a short interval after photoexcitation and a long decaying part that extends up to tens of milliseconds. These decay behaviors look different depending on the mesoscopic structures and the perovskite morphology formed therein, as seen from their Scanning Electron Microcopy images and X-ray diffraction patterns. The decay part can be fitted with a three-exponential decay, which reflects different kinetics of electrons in the perovskite/TiO_2_ layer. On the other hand, the rising part must be fit by a decay equation derived by employing the convolution theorem, where the rising part can be assigned to the electron transport process inside the perovskite layer and the decaying part can be assigned to electron back-transfer. The characteristics can be then understood by considering the effect of crystal defects and trap states in the perovskite grains and perovskite interface with its transport layer, which is TiO_2_ in this study. Although the TPV decay occurs in a time range much longer than the primary process of photoexcitation as commonly observed in transient photoluminescence spectroscopy, the processes involved in this TPV strongly correlates with the performance of these perovskite solar cells.

## Introduction

Since the first reports by Gratzel et al. and Snaith et al., remarkable improvements in the power conversion efficiency of perovskite solar cells (PSCs) have been reported by several groups^1–5^. Nowadays, perovskite solar cell (PSC) efficiency can reach up to more than 20%, which significantly compete with their counterpart silicon solar cells. Nevertheless, the reproducibility and stability of PSCs remain to be the main problem for mass production. Although these perovskites are formed based on uncomplicated stoichiometry rules, producing reproducible perfect perovskite layers is still a challenging task. The problem lies in the fact that the crystallization occurs through wet chemical processes in a very short time. The crystallization time for these perovskites is much shorter than that of other crystalline materials, such as silicon-based crystals. As a result, various crystal defects may be formed, which can reduce the crystal stability and degrade the performance of the solar cells.

In addition, the crystal binding in these perovskites is dominated by ionic bonding characters, even though valence bonding characters are also found to contribute partially^6,7^. This is fairly different from silicon crystals, which are built entirely by covalent bonding. Therefore, perovskite crystals are more sensitive to environments, such as a humid-atmosphere, oxygen contaminated atmosphere, and temperature. Unfortunately, crystal defects can still grow further even after the formation of the perovskite layer in the fabrication process, such as during the transport layer and metal contact deposition, and during storage or even during cell operation^8^.

Various kinds of possible crystal defects in bulk perovskite crystal have been predicted, such as vacancies, interstitial defects, lattice distortion by accumulated charges, and dissolved impurities^9–12^. Vacancy or interstitial defects may also be formed at the surfaces of domain boundaries or layer interfaces, which are also commonly found in most crystalline materials. Those defects may create shallow and deep levels in the bulk bandgap. In some recent years, several reports are showing the improvement in the PSC stability and performance by the addition of additive or passivation molecules with the purpose to reduce or passivate the defects^3–5,13–17^. However, it is still necessary to gain more understanding in the detailed mechanism and extent of these defects affecting the PSC performance. For answering this question, it may require a detailed study on the entire photovoltaic processes, from photoexcitation until charge carrier extraction, and the effect of defects on those processes. However, this is not easy because there is no existing technique to characterize both crystal defects and photovoltaic properties simultaneously. Several methods have been reported to indirectly verify the presence of defect or trap states and their effects on the charge carrier transports and solar cell performance, such as impedance spectroscopy, microwave conductivity, transient photocurrent, and transient photoluminescence^9,18,30^. Photogeneration of charge carriers in these perovskites is very effective, and free electrons and holes are generated spontaneously after photoexcitation. Nevertheless, the accompanying charge carrier transport and extraction processes also crucially affect the performance of the solar cells^16,19^. Defects in the perovskite layer, either inside the grains or at the grain surfaces, may affect both charge carrier transport and charge carrier extraction processes.

Using transient photoluminescent spectroscopy, Stranks et al. found that the evolution of photoexcited species is significantly affected by electronic traps at low excitation fluence conditions^20^. However, at high excitation fluence, when most trap states have been filled, the evolution is dominated by radiative bimolecular recombination. The role of defect states in the recombination of hot carriers in these perovskites has also been reported using transient photoluminescence spectroscopy and transient absorption spectroscopy^21,22^. The presence of hot carriers and their recombination are very important for improving perovskite-based devices. Moreover, the role of these hot carriers has also important implications in other applications of these perovskites, such as light-emitting diodes (LEDs) and lasing devices^23–26^. However, those processes occur shortly after photoexcitation in the sub-nanosecond regime under high-intensity photoexcitation. Therefore, it may raise a question on whether those processes crucially are responsible directly to the variation of solar cell performance commonly observed in the fabrication process^27^. Indeed, we may assume that these perovskite materials also obey the general reciprocity relation in maximizing the performance of LEDs and solar cells, as commonly observed in organic semiconductor and semiconducting polymer materials^28–30^. The charge carriers involved in the photovoltaic process are the surviving charge carriers from the fast radiative and non-radiative recombination processes. In PSCs, the entire photovoltaic processes may even extend beyond the order of microseconds^31,32^. In this paper, we report experimental results on the kinetics of charge carrier transport and extraction by performing transient photovoltage (TPV) measurements, in order to see the correlation between electronic processes, which can be affected by defect states caused by mesoscopic structural characteristics of the perovskite layers, and the PSC performance.

## Results

**Figure 1:**
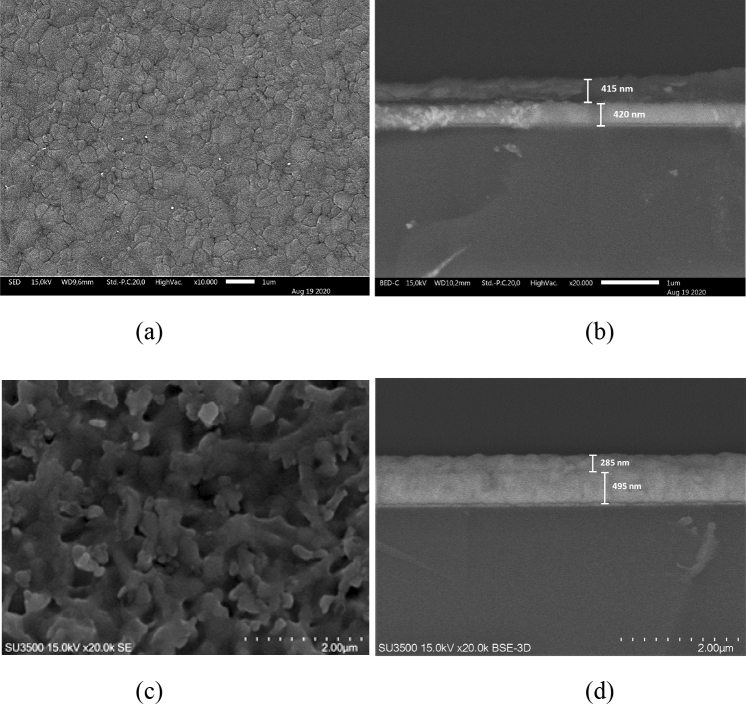
The SEM images of the (**a**) surface and (**b**) cross-section of perovskite nanocrystals on the mp-TiO_2_#A layer and the (**c**) surface and (**d**) cross-section on the mp-TiO_2_#B layer. These cross-section images were taken by Back-Scattered Electron (BSE) measurement mode. The multiplication labels dan scale indicators are different in those images.

**Figure 2:**
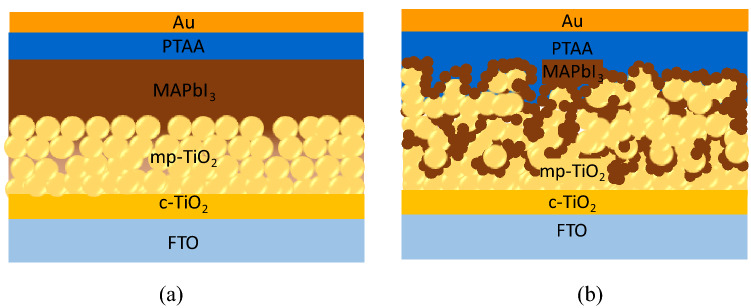
Illustration of mesoscopic structures of (**a**) the cell-A and (**b**) the cell-B (the figures were adapted and modified from Xiao et al.^44^. The brown color schematically represents the perovskite crystals, while the yellow color represents the TiO_2_ nanoparticles (see the text for a detailed explanation of this illustration).

The PSC samples have a standard cell structure consisting of mesoporous TiO_2_ and MAPbI_3_ layers, that is, with a multi heterolayer structure of FTO/c-TiO_2_/mp-TiO_2_/MAPbI_3_/PTAA/Au configuration, where MAPbI_3_ (methylammonium lead triiodide) is the perovskite layer. All solar cell samples were prepared using the same materials and processes except for their mesoporous layers (mp-TiO_2_). The first cell type (cell-A) samples have their mp-TiO_2_ layers that were prepared from diluted TiO_2_ pastes with ethanol at a 1:2 vol%. The second cell type (cell-B) samples have their mp-TiO_2_ layers that were prepared from diluted TiO_2_ pastes in ethanol with 1:16 wt%. The resulting perovskite layers represent different mesoscopic structural characteristics, as seen from their Scanning Electron Microscopy (SEM) images. Figure 1a,b show the SEM images of surface and cross-section for MAPbI_3_ perovskite deposited onto the type A of the mp-TiO_2_ layer (mp-TiO_2_#A). Figure 1c,d show the SEM images for MAPbI_3_ perovskite on type B of the mp-TiO_2_ layer (mp-TiO_2_#B). It is apparent that the formed perovskite nanocrystals are partially adsorbed inside the mp-TiO_2_#A layer but the remaining perovskite crystals form a very thin layer on the top of it. In contrast, due to larger pores in the mp-TiO_2_#B layers, the formed perovskite nanocrystals are entirely absorbed into the mp-TiO_2_#B layer without forming a thin layer on top of it. Hence, the cell-A resembles to form a common mesopore structure, while the cell-B forms a scaffold-like structure (Fig. 2).

The cross-section SEM images in Fig. 1b,d, which were taken from the Back-Scattered Electron (BSE) measurements, show almost the same total thicknesses in those samples. The thickness of those layer structures, which are consisting of the FTO layer, TiO_2,_ and perovskites, are about 850–900 nm. The FTO layer is seen as a brighter white color region with the thickness to be around 400–500 nm. The upper layer is seen as then TiO_2_ and perovskite layer with the thickness around 400–450 nm. The TiO_2_/perovskite layer appears slightly thicker in the mp-TiO_2_#A sample rather than in the mp-TiO_2_#B sample, namely ~ 400 nm vs. ~ 300 nm, respectively. The presence of the perovskite layer deposited on the top of the TiO_2_ layer in the mp-TiO_2_#B sample thus increases the total thickness. It is then quite reasonable to find a stronger UV–Vis absorption in the TiO_2_#A sample in comparison to the TiO_2_#B sample, as seen in Supplementary Fig. S1.

It should be also noted that those samples show perfect perovskite crystal formation as seen from their XRD patterns, which can be seen in Supplementary Fig. S2. Both samples show intense diffraction peaks at 14.2°, which is originated from (110) plane, and a medium intense diffraction peak at 28.6°, which is originated from (220) plane. This indicates that the formed perovskite crystals possess a tetragonal structure, which is in agreement with other reports elsewhere^33,34^. While the XRD patterns of those samples are the same, the widths of their XRD peak are apparently different. The full width at half maximum (FWHM) values of (110) peak is 0.11° and 0.15° for perovskite on the mp-TiO_2_#A layer and the mp-TiO_2_#B layer, respectively. The FWHM value is often to be used for estimating the crystallite size by employing the Debye Scherrer formula^35,36^. The calculated crystallite size for perovskite on the mp-TiO_2_#A layer is thus about 75 nm, while for the perovskite on mp-TiO_2_#B is about 56 nm. The perovskite on the mp-TiO_2_#A has a larger perovskite because perhaps the crystal can grow faster in a flat layer formation rather than in a curved layer formation that occurs in the mp-TiO_2_#B sample.

As also seen in those SEM images, the FTO thickness looks slightly different, which is likely due to unexpected thickness variation in different batch production of received FTO from the maker. Those FTO were used as received without any special pre-treatment except a simple cleaning process just by using an ethanol/acetone cleaning solution. However, we consider that those FTO thickness differences in this case will not affect the entire photovoltaic properties of the fabricated solar cells.

**Figure 3:**
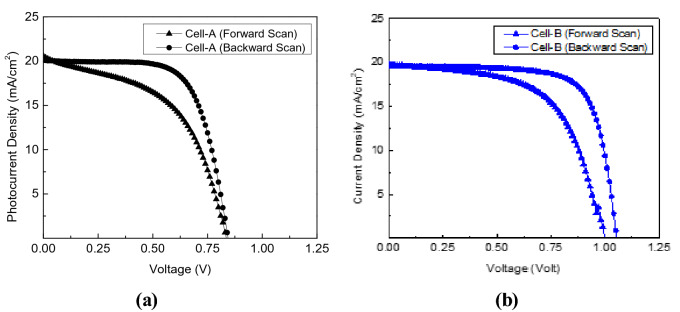
J–V characteristics of (**a**) the cell-A and (**b**) the cell-B samples.

Figure 3a,b show the photovoltaic properties (*J–V* curve) of the cell-A and cell-B samples, respectively. The cell-A seems to exhibit poorer performance than the cell-B. The cell-A yields *J*_*sc*_, *V*_*oc*_, *FF,* and *PCE* values of 20.0 mA/cm^2^, 0.84 V, 68.1%, and 11.5%, respectively. However, the cell-B can produce much better performance, namely 19.6 mA/cm^2^, 1.05 V, 73.4%, and 15.1% for those values, respectively. In order to confirm the validity of this *J–V* measurement results, the cell-B sample has been also measured its External Quantum Efficiency (EQE). As seen in Supplementary Fig. S3, the average EQE is about 75% in the wavelength range of 350–750 nm. This average EQE is slightly larger than the previous result for an inverted perovskite structure that yields around 12.5% solar cell efficiency^17^.

**Figure 4:**
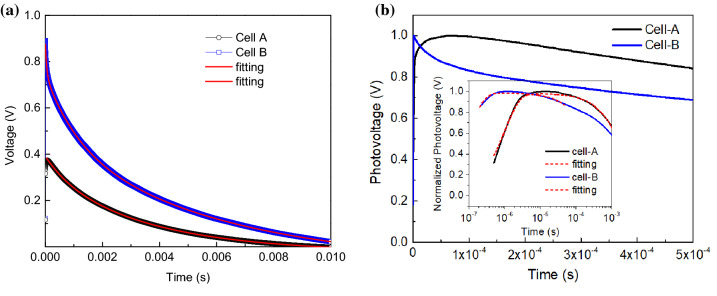
(**a**) Transient photovoltage (TPV) decay of the cell-A and the cell-B. The thin red lines are the fitting lines with a function of three-exponential decay Eq. (1). (**b**) The same TPV curve but shown in a short time interval after laser pulse excitation in both linear and logarithmic scale (inset figure) of the x-axis. The decay curves are plotted on a normalized scale for clarity. The thin dash lines (red color) in the inset figure are the fitting lines (see “Discussion” section for the detail).

Figure 4 shows the TPV decays measured from those cell-A and cell-B samples. The decays are apparently different although they can be fitted by a three-exponential decay function given by:

1 $$V\left( t \right) = V_{1} e^{{ - {\raise0.7ex\hbox{$t$} \!\mathord{\left/ {\vphantom {t {\tau_{1} }}}\right.\kern-\nulldelimiterspace} \!\lower0.7ex\hbox{${\tau_{1} }$}}}} + V_{2} e^{{ - {\raise0.7ex\hbox{$t$} \!\mathord{\left/ {\vphantom {t {\tau_{2} }}}\right.\kern-\nulldelimiterspace} \!\lower0.7ex\hbox{${\tau_{2} }$}}}} + V_{3} e^{{ - {\raise0.7ex\hbox{$t$} \!\mathord{\left/ {\vphantom {t {\tau_{3} }}}\right.\kern-\nulldelimiterspace} \!\lower0.7ex\hbox{${\tau_{3} }$}}}} ,$$

where *V*_*i*_ and *τ*_*i*_ are the amplitude and decay constant of the *i*-th decay component (*i* = 1, 2, 3). The decay parameters obtained from the fitting for both cells are shown in Table 1. The time constants of the fast decay component in both cells ($\tau_{1}^{A}$ and $\tau_{1}^{B}$) are in the same time order of microseconds. However, $\tau_{1}^{A}$ is about 18 times greater than $\tau_{1}^{B}$. The mid-decay time constants of both cells ($\tau_{2}^{A}$ and $\tau_{2}^{B}$) are also in the same time order of milliseconds, where $\tau_{2}^{A}$ is just several times larger than $\tau_{2}^{B}$. The third decay component of the cell-A ($\tau_{3}^{A}$) shows a prolonged decay almost 10 ms, which is almost twice the cell-B decay. However, it is interesting to notice that, by noting the *%A* values in Table 1, the decay portion is dominated by the second decay component ($\tau_{3}^{A}$) in the cell-A case, but it is dominated by the slowest decay component ($\tau_{2}^{B}$) in the cell-B case. The *%A* value of each decay component is the ratio of the initial voltage of the *i*-th decay component *V*_i_ to the total initial voltage *V*.

**Table 1:** Solar cell performance parameters determined from the *J–V* curves and fitting results obtained from the TPV decay shown in Figs. 3 and 4.

	Cell A			Cell B		
Solar cell performance						
	*J*_*sc*_ (mA/cm^2^)	*V*_*oc*_ (V)	PCE (%)	*J*_*sc*_ (mA/cm^2^)	*V*_*oc*_ (V)	PCE (%)
	20.0	0.84 V	11.5%	19.6	1.05 V	15.1%

Decay fitting results (by three exponential decays)						
	Fast decay $(\tau_{1}^{A} )$	Mid-decay $(\tau_{2}^{A} )$	Slow decay $(\tau_{3}^{A} )$	Fast decay $(\tau_{1}^{B} )$	Mid-decay $(\tau_{2}^{B} )$	Slow decay $(\tau_{3}^{B} )$
*V*	0.07 V	0.29 V	0.06 V	0.13 V	0.27 V	0.55 V
*%A*	16.7%	69.0%	14.2%	13.7%	28.4%	57.9%
*τ*	790.3 × 10^–6^ s	3.1 × 10^–3^ s	9.2 × 10^–3^ s	50.8 × 10^–6^ s	1.1 × 10^–3^ s	5.9 × 10^–3^ s

Another interesting feature appears in the TPV decays is in their rising part, which apparently has a quite different rising shape, as seen in Fig. 4b. For the cell-A case, the curve rises slowly until reaching its maximum after pulse photoexcitation. However, in the cell-B case, the curve rises sharply to reach its maximum. It can be seen more clearly when the *x*-axis is plotted on the logarithmic scale, as shown by the inset figure in Fig. 4b. The maximum initial decay amplitude of the cell-A is smaller than that of the cell-B seems consistent to be consistent with the fact that cell-A has a lower cell performance.

**Figure 5:**
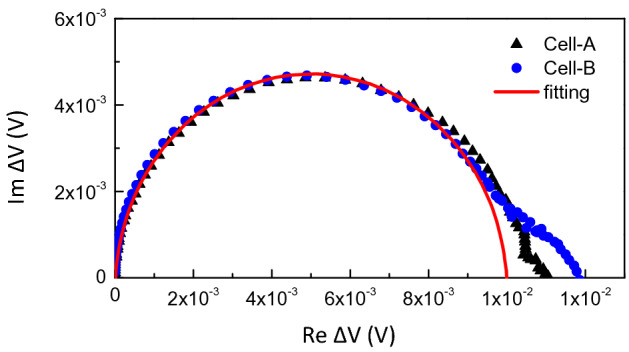
The Nyquist plot obtained from the IMVS measurements of the cell-A and cell-B. The red dash line is a line just for showing a half circle line shape.

Figure 5 shows the Nyquist plots measured from the cell-A and cell-B. Because the measurements were done by using a lock-in amplifier, the obtained IMVS data can be then presented only in voltage (V) and not in impedance (Ohm). However, we may still expect that the plot shown in Fig. 5 has similar features as their actual impedance characteristics, such as its characteristics frequency. As seen in the figure, although both show almost similar semicircle shape, the characteristic frequency assigned from the data point at the maximum of the semicircle are 281 Hz and 126 Hz for the cell-A and cell-B, respectively.

## Discussion

It is obvious that there are some remarkable differences in the photovoltaic properties and TPV decay in those PSC samples. Because all raw materials and fabrication processes were the same for those PSC samples except in their mp-TiO_2_ layers, we may consider that there is a difference in the mesoscopic structural characteristics of their perovskite layer and its interface with the mp-TiO_2_. However, it should be noted that the present work is not aimed to determine which the best structure for producing high conversion efficiency. It should be also noted that we did not add additives or passivation substances to ensure that the defects are intrinsically originated from the perovskite crystals.

**Figure 6:**
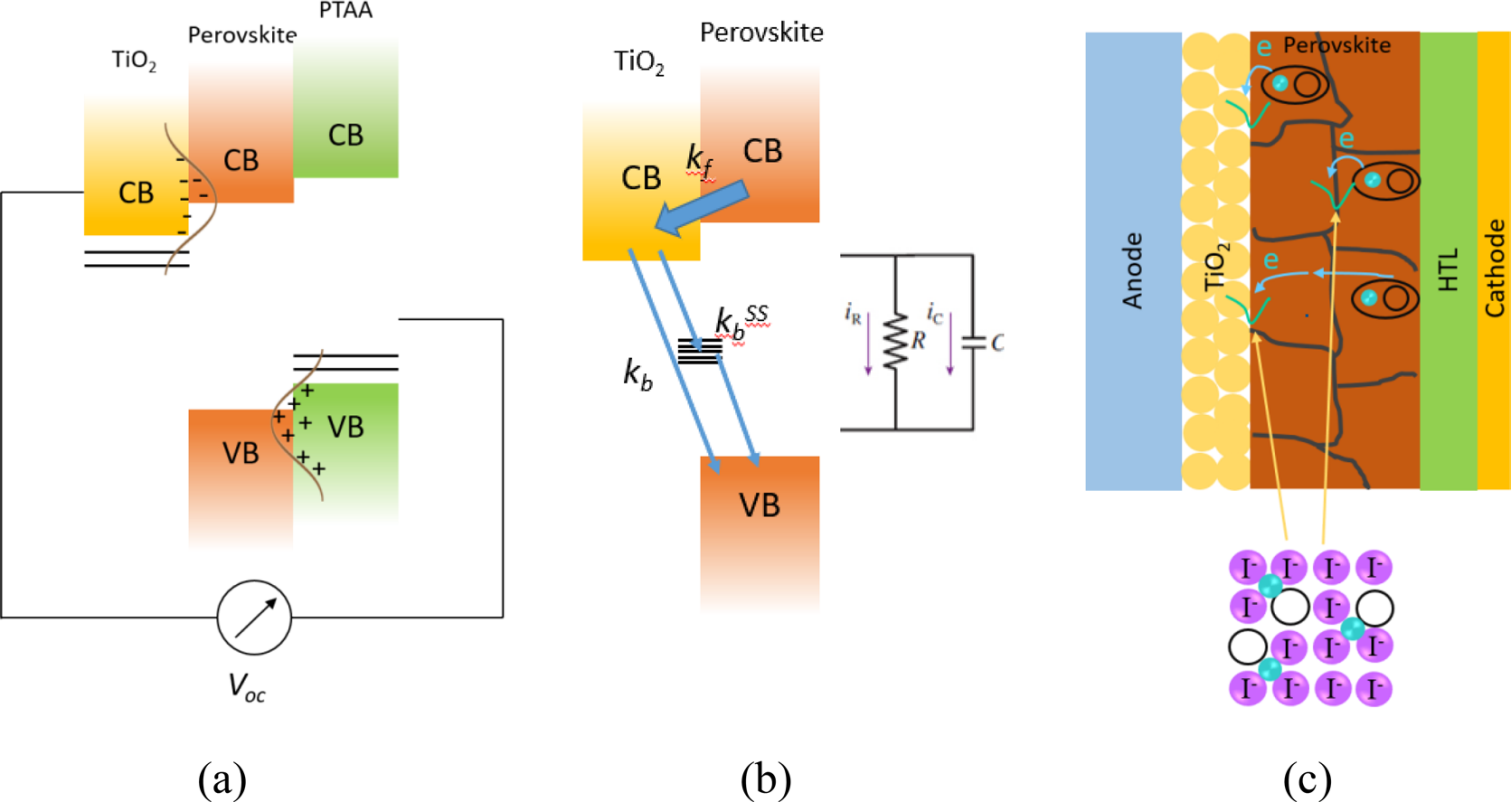
(**a**) Charge accumulation at the interface and the formation V_oc_. (**b**) Electron accumulation and its subsequent relaxation back to the perovskite layer. (**c**) Electron recombination at the TiO_2_/perovskite interface and trapping-detrapping at the grain boundaries.

Firstly, we should recall the open-circuit voltage in a solar cell consisting of amorphous active material, where there are disorder distributions near the bottom of the conduction band and the top of the valence band, as illustrated in Fig. 6a In such a case, the open-circuit voltage is given by^37,38^:

2 $$qV_{OC} = E_{g} - \frac{{\sigma_{n}^{2} + \sigma_{p}^{2} }}{{2k_{B} T}} + k_{B} T \ln \left( {\frac{np}{{N_{e} N_{v} }}} \right),$$

where *σ*_*n*_ (*σ*_*p*_) is the width of the state distribution at the lowest conduction band (the upmost valence band), while *n* (*p*) is the electron (hole) density, and *N*_*c*_ (*N*_*v*_) is the density states of the conduction band (valence band). A broader distribution results in smaller *V*_*oc*_. Because perovskite is a crystalline material, the broad state distribution may be due to the presence of defect or surface states, which may also behave as trap states. Because *V*_*oc*_ of the cell-A is smaller than that of the cell-B, we may suggest that there are more defects or surface states at the TiO_2_/perovskite interface in the cell-A.

Because TPV measurement is conducted under open-circuit conditions, the TPV decay represents only the recombination of accumulated charge carriers at the interface of the charge transport and perovskite layers, as illustrated in Fig. 6a. This situation is quite different from TPC measurement, which also involves the extraction of charge carriers accumulated at the transport layers because the cell is operating under short-circuit condition. However, those processes are entirely different from what happened in Transient Photoluminescence (TPL) measurement, where the decay is originated dominantly from bulk recombination of charge carriers just inside the active layer without requiring charge transport to the layer interface. In general, as primary processes after photoexcitation, the recombination process may occur through radiative recombination, Shockley–Read–Hall (SRH) recombination (which involves trap states or defect states), and Auger recombination. The effective lifetime in bulk (*τ*_*b*_) is given by^39^:

3 $$\frac{1}{{\tau_{b} }} = \frac{1}{{\tau_{rad} }} + \frac{1}{{\tau_{SRH} }} + \frac{1}{{\tau_{Aug} }},$$

where *τ*_*rad*_, *τ*_*SRH*_, and *τ*_*Aug*_ are the time constant of radiative, SRH and Auger recombinations. For nanocrystalline structures, the excited state or charge carrier diffusion length is comparable to the particle size, and the recombination at the surface becomes more dominant. In such a case, the effective lifetime (*τ*) can be written as^39^:

4 $$\frac{1}{\tau } = \frac{1}{{\tau_{b} }} + \frac{1}{{\tau_{s} }},$$

where *τ*_*s*_ is the time constant of surface recombination. The diffusion of electrons and holes might be involved in both SRH recombination and surface recombination. However, the diffusion length is very small compared to the grain size or thickness of the perovskite layer in these PSC samples^40^. On the other hand, photoluminescence decays in this perovskite have been much reported in the literature with the lifetime constant in a few nanoseconds^20,26,30,40^. This time range is even much shorter than the observed fastest decay component in the present TPV decay. Therefore, we may suppose that this perovskite also follows the general principle where photovoltaic processes involve merely the surviving charge carriers produced from those very fast processes (primary processes) observed in TPL. The photovoltaic characteristics are then determined by the recombination of those charge carriers happening at a longer time range.

Generally. the observed three decay components in the TPV curve can be assigned to three different dynamics of charge carriers. We may then suppose that the fastest decay is related to direct recombination of the accumulated electrons, while the slower decays may be related to the charge carrier recombination assisted by trap states or the recombination of long-lived charge carriers. If we consider that in this TPV the trap-state-assisted recombination is akin to SRH recombination taking place on the surface states at the TiO_2_/perovskite interface, the decay time constant will be given by^28^:

5 $$\tau_{n,SRH} = \frac{1}{{B_{n} N_{t} }} = \frac{1}{{v_{n} \sigma_{n} N_{t} }},$$

where *B*_*n*_ is the trapping coefficient for electrons, *v*_*n*_ is the mean thermal velocity of the electrons, *σ*_*n*_ is the capture cross-section of defects or traps for electrons, and *N*_*t*_ is the trap state concentration. Consequently, the decay time constant is shorter for a larger number of trap states and hence implies a poorer cell performance. However, this argument seems contrary to the present TPV data, where the cell with a shorter decay time correlates to a better cell performance, as observed in the cell-B. The cell-A that exhibits poorer cell performance shows longer time decay.

The interpretation of TPV decay is often ambiguous, where the interpretation for the origin of the time constant has long been a controversial issue. The interpretation may largely vary case by case, particularly when the observed time constant is much longer than recombination processes observed in TPL measurement. Kiermasch et al. have tried to clarify this problem and they showed that TPV decay in PSC is likely governed by the capacitive electrode associated with accumulated charges from the active layer^27^. This also implies that the observed TPV decay cannot be directly assigned to the charge carrier lifetime in the perovskite layer, as in TPL spectroscopy. Therefore, the TPV decay is rather to be related to the kinetics of the accumulated electrons at the TiO_2_/perovskite interface or holes at the PTAA/perovskite interface. Immediately after the pulse photoexcitation, the photogenerated charge carriers in the perovskite layer move to the transport layers through either diffusion current or drift current. The cell voltage then changes with time as given by $$V\left( t \right) = {\raise0.7ex\hbox{${\Delta Q\left( t \right)}$} \!\mathord{\left/ {\vphantom {{\Delta Q\left( t \right)} C}}\right.\kern-\nulldelimiterspace} \!\lower0.7ex\hbox{$C$}}$$, where $$\Delta Q\left( t \right)$$ (= *e* Δ*n(t)* = *e* Δ*p(t)*) is the total electrons accumulated at the TiO_2_ layer or holes at the PTAA layer, and *C* is the cell capacitance. In contrast to TPC, the decay of these accumulated charge carriers is not due to the charge carriers extraction to the external circuit but solely due to internal recombination as illustrated in Fig. 6b. The charge transfer rate of electrons from the perovskite layer to the TiO_2_ layer (*k*_*f*_) is supposed to be very high and thus very small time constant. However, there might be some back-transfer pathways for those accumulated electrons, with very low transfer rates, either by a direct pathway from TiO_2_ to the perovskite layer or via surface trap states. Such a situation may be represented equivalently as a parallel *RC* circuit, as illustrated in the inset of Fig. 6b. During ultrashort pulse laser exposure, the photogenerated electrons quickly flow filling up the cell. After the pulse laser exposure, the capacitor then slowly discharges the accumulated electrons through the resistor *R*. A smaller back transfer rate can be associated with a larger resistance *R*. This may qualitatively explain the observation slow decay part in TPV.

The width of the laser pulse used in this work was a few nanoseconds, which is much shorter than the measured TPV response. The kinetics of the electron population on the TiO_2_ side can then be written as:

6 $$e\frac{\partial \Delta n\left( x \right)}{{\partial t}} = \frac{{\partial J_{n} }}{\partial x} - eR,$$

where the left side represents the change of accumulated electron number (*Δn*) with time, while the first term on the right side is related to electron flow inside the TiO_2_ layer (*J*_*n*_ is current density distribution inside the mp-TiO_2_ layer), and the second term on the right side is related to the recombination rate (*R*) of those accumulated electrons. We assume that the photogeneration charge carrier can be neglected because the incoming photon energy is not sufficient to produce photoexcitation in TiO_2_. We may also assume that the short pulse photoexcitation can supply plenty of electrons for filling the TiO_2_ layer uniformly. In such a circumstance, the first term on the right side of Eq. (6) can be neglected.

The second term in the right side of Eq. (6) may be comprised of some possible recombination processes, such as the band-to-band recombination inside the TiO_2_ layer, direct back transfer from the conduction band of TiO_2_ to the valence band, and trap/defect-state-assisted back transfer from TiO_2_ to the valence band of the perovskite layer. The recombination is either monomolecular or bimolecular recombination. However, Stranks et al*.* have shown that the bimolecular recombination occurs only at high-fluence photoexcitation, which is not the case for the present work^20^. The pulse laser energy used in this present work was only tens of microjoules per pulse. If the recombination rate *R* is simply proportional to *n*/*τ*_*rec*_, where *τ*_*rec*_ is the time constant of the recombination rate, the kinetic rate of recombination at the TiO_2_/perovskite interface becomes:

7 $$\frac{\partial n}{{\partial t}} = - \frac{n}{{\tau_{rec} }}.$$

Because the pulse laser width is much shorter than the TPV decay, we may expect the solution of Eq. (7) will be a simple exponential function in the form of $\Delta n\left( t \right) = \Delta n_{0} e^{{ - \frac{t}{{\tau_{rec} }}}}$, where *n*_*0*_ is the initial electron concentration supplied from the perovskite layer after photoexcitation. Consequently*,* the TPV decay (*ΔV*(*t*)) would be just a simple exponential^41^. However, this cannot explain the appearance of the rising part with different profiles in the present TPV decays.

We then could consider that $$\Delta n_{0}$$ is changing with time, which represents the supply of electrons from the perovskite layer that is affected by the transport history of those electrons themselves. The above solution then should be modified by a convolution of $$\Delta n_{0} \left( t \right)$$ and the exponential decay $$e^{{ - \frac{t}{{\tau_{rec} }}}}$$. Based on the convolution theorem, for $$t \ge 0,$$ the solution for *V*(*t*) = $$\left( {{\raise0.7ex\hbox{$e$} \!\mathord{\left/ {\vphantom {e C}}\right.\kern-\nulldelimiterspace} \!\lower0.7ex\hbox{$C$}}} \right) n_{rec} \left( t \right) \times n_{t} \left( t \right)$$, where $$n_{rec} \left( t \right) \propto e^{{{\raise0.7ex\hbox{${ - t}$} \!\mathord{\left/ {\vphantom {{ - t} {\tau_{rec} }}}\right.\kern-\nulldelimiterspace} \!\lower0.7ex\hbox{${\tau_{rec} }$}}}}$$ and $$n_{t} \left( t \right) \propto e^{{{\raise0.7ex\hbox{${ - t}$} \!\mathord{\left/ {\vphantom {{ - t} {\tau_{t} }}}\right.\kern-\nulldelimiterspace} \!\lower0.7ex\hbox{${\tau_{t} }$}}}}$$, is given by

8 $$V\left( t \right) = V_{0} \left( {\frac{{e^{{{\raise0.7ex\hbox{${ - t}$} \!\mathord{\left/ {\vphantom {{ - t} {\tau_{t} }}}\right.\kern-\nulldelimiterspace} \!\lower0.7ex\hbox{${\tau_{t} }$}}}} - e^{{ - {\raise0.7ex\hbox{$t$} \!\mathord{\left/ {\vphantom {t {\tau_{rec} }}}\right.\kern-\nulldelimiterspace} \!\lower0.7ex\hbox{${\tau_{rec} }$}}}} }}{{{\raise0.7ex\hbox{$1$} \!\mathord{\left/ {\vphantom {1 {\tau_{rec} }}}\right.\kern-\nulldelimiterspace} \!\lower0.7ex\hbox{${\tau_{rec} }$}}{\raise0.7ex\hbox{${ - 1}$} \!\mathord{\left/ {\vphantom {{ - 1} {\tau_{t} }}}\right.\kern-\nulldelimiterspace} \!\lower0.7ex\hbox{${\tau_{t} }$}}}}} \right).$$

The parameter *τ*_t_ is the time constant of electron transport inside the perovskite layer and *τ*_rec_ is electron back-recombination that is illustrated by Fig. 6b, and *V*_*0*_ is the initial photovoltage. The curve of this solution consists of a rising part in the earlier time after photoexcitation, which is then followed by a decaying part. The TPV decays in Fig. 4 were then fitted by Eq. (8) for the time interval between 0.1 and 100 µs, which covers the rising part and the beginning part of the fastest decay component. The curve lines of the fitting results are also shown in that figure. The fitting results yield *τ*_*t*_ = 1.03 µs and *τ*_*rec*_ = 1.96 ms for the cell A, while *τ*_*t*_ = 0.10 µs and *τ*_*rec*_ = 0.37 ms are obtained for the cell-B. The cell-A that exhibits poorer solar cell performance shows a longer *τ*_*rec*_, which can be associated with a greater number of surface defect states and deep level defect states in the cell-A. Therefore, electrons quickly trapped at the defect surface states and stay for a while at those states before back transferred into the perovskite valence band. Here, the recombination rate via deep level states is commonly much slower than direct recombination, namely *k*_*b*_^*SS*^ < *k*_*b*_, as illustrated in Fig. 6b. Using capacitor pictorial understanding, this means that the discharging process will take a longer time and hence results in longer time decay in the cell-A. On the other hand, faster time decay can thus be correlated with less defect surface states and better solar cell performance. Such recombination and trapping-detrapping processes may also take place not only at the perovskite/TiO_2_ interface but also in grain boundaries between perovskite crystals, as illustrated in Fig. 6c.

**Figure 7:**
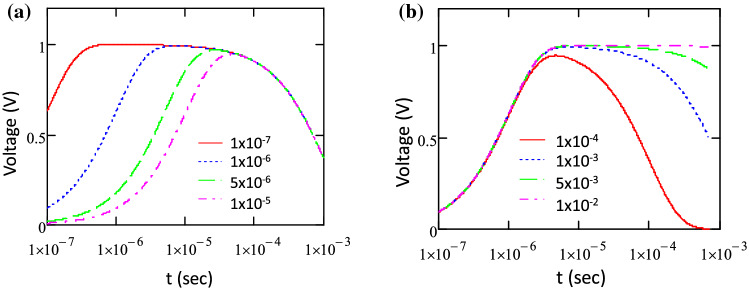
The simulation of Eq. (8) calculated for (**a**) various τ_t_ value ranging from 1 × 10^–7^ up to 1 × 10^–5^ with τ_rec_ = 1 ms and (**b**) τ_rec_ from 0.1 ms up to 10 ms with τ_t_ = 1 × 10^–6^ s.

While *τ*_*rec*_ has a major effect on the decaying part, the decay constant *τ*_*t*_ has a more noticeable effect on the rising part of the curve. Figure 7a shows a simulation of the fitting function Eq. (8), which indicates a slower rising part of the curve with the increase of *τ*_*rec*_. These simulation curves were calculated for *τ*_*rec*_ = 1 ms and various transport time constant *τ*_*t*_ ranging from 1 × 10^–7^ up to 1 × 10^–5^ s. On the other hand, Fig. 7b shows that the decaying part extending longer with the increase of *τ*_*rec*_, as seen here with the increase of *τ*_*rec*_ from 0.1 ms up to 10 ms, which were calculated for *τ*_*t*_ = 1 × 10^–6^ s. The longer rising part observed in the cell-A (Fig. 4) therefore corresponds to a longer *τ*_*t*_, which may then be associated with a longer transport time needed for electrons reaching and populating the TiO_2_ layer. The diffusion coefficient and charge carrier mobility in this perovskite have been reported to be around 0.3 cm^2^ s and 1–100 cm^2^/Vs, respectively^32^. One can then estimate that the transit time of charge carriers inside this perovskite layer using the following relationships for drift current: $$t_{tr} = {\raise0.7ex\hbox{${d^{2} }$} \!\mathord{\left/ {\vphantom {{d^{2} } {\mu V_{oc} }}}\right.\kern-\nulldelimiterspace} \!\lower0.7ex\hbox{${\mu V_{oc} }$}}$$, where *d* is the layer thickness, *µ* is the electron mobility and *V*_*oc*_ is the open-circuit voltage; and the following relationships for diffusion current : $$t_{tr} = {\raise0.7ex\hbox{${d^{2} }$} \!\mathord{\left/ {\vphantom {{d^{2} } D}}\right.\kern-\nulldelimiterspace} \!\lower0.7ex\hbox{$D$}}$$, where *D* is the diffusion constant. Therefore, we may roughly estimate those transit times to be several nanoseconds to hundreds of nanoseconds, which overlaps with the transport time constant *τ*_*t*_ values obtained from this experiment.

A larger transport time *τ*_*t*_ value in the cell-A may be caused by several possibilities. For instance, it may be simply due to a larger thickness of the perovskite layer in the cell-A sample, as seen in the SEM image. Otherwise, it may be also due to a larger effect of trapping-detrapping of electrons during their migration inside the perovskite layer to the TiO_2_ layer. Besides trapping-detrapping via bulk defects, trapping-detrapping may also occur at grain boundaries, which are much more apparent in the perovskite layer of the cell-A. The trapping-detrapping process slows down the electron transport speed and decreases the diffusion/drift constant. The trapping-detrapping may cause a filling process at the beginning of the time after photoexcitation, as observed by Stranks et al.^20^. Particularly, in the case of the cell-A, the formation of a thick perovskite layer in a short time duration is prone to interface delamination cracking. Such cracking may occur slowly due to delayed solvent evaporation and recrystallization leading to a denser perovskite layer. However, due to the lack of molecular bonding or molecular anchoring between the TiO_2_ and perovskite, this perovskite layer may randomly undergo delamination cracking from the TiO_2_ surface. In such a case, a portion of photogenerated electrons will be trapped on the surface of those delaminated perovskite layers and recombine without contributing to the photovoltaic output of the solar cell. However, some trapped electrons may eventually gain energy to escape and finding a path to reach the TiO_2_ layer. However, such a situation causes a large extraction charge carrier extraction loss leading to poor solar cell performance.

**Figure 8:**
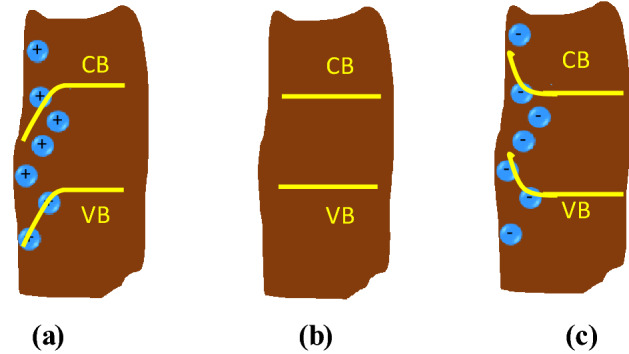
Band bending on the surface of perovskite grain caused by the spatial distribution of (**a**) positive charge due to I^-^ vacancies, (**b**) neutralized surface states, after being filled by photogenerated electrons, and (**c**) excess or accumulated electrons at the surface of perovskite grains or interfaces. the cell-B (the figures were adapted and modified from Wang et al.^32^).

In addition, it is also worth considering the effect of defect/surface states at the TiO_2_/perovskite interface. The perovskite crystal is constructed from ionic bonds of Pb^2+^ and MA^+^ cations and I^-^ anions, so there are unbalanced charges on the surfaces of grains or interface. Iodide vacancies (*V*_*I*_) may be intrinsically present in the perovskite layer formation, that are formed during the fabrication, or due to ionic migration from their original sites during cell storage or operation. In both cases, the surface becomes more positively charged as the MA^+^ and Pb^2+^ cations have lost their valence electrons to I^-^ anions, where this situation may lead to a downward band bending at the surface/interface^10,32^. Immediately after photoexcitation, photogenerated electrons partially fill up those trap states and gradually reduce the band bending. However, as the electrons accumulate at the surface, the band bends upward, leading to a potential barrier for electron transport, as illustrated in Fig. 8. Therefore, we may suppose that the current flow to the TiO_2_ layer would decrease exponentially, which becomes the reasoning basis for choosing the exponential form of the *n*_*0*_(*t*) above.

It is then also relevant to compare these TPV characteristics with the cell impedance characteristics, which is commonly measured in the range of milliseconds up to seconds of time periodicity^32^. As shown by the IMVS measurement results in Fig. 5, the cell-A and cell-B have characteristics frequencies at about 281 Hz and 126 Hz, which can then correspond to the decay time constants of 3.6 ms and 7.9 ms, respectively. These decay time constants almost equal and correlate to the dominant decay component in the TPV of each cell, which is the second decay component of the cell-A and the slowest decay component in the cell-B. Indeed, the origin of this semicircle still cannot determine clearly at the present stage because the semicircle is less affected by the type of TiO_2_ layer. Gonzalez-Pedro *et al*. have shown, from impedance spectroscopy measurements, that the semicircle at high-frequency is related to the transport in the spiro-MeOTAD layer while the semicircle at low-frequency is related to the transport–recombination process in the TiO_2_/perovskite layer^42^. Considering their results, because the semicircle in our IMVS data is from ~ 1 Hz up to ~ 10 kHz, we may consider that the observed semicircle data may be also related to the transport–recombination process in TiO_2_/perovskite layer. The transmission line characteristic is not observed because in this IMVS measurement the cell is under the open-circuit condition and the injected electrons-holes are from inside the cell through photo-excitation. In such a case, the recombination at the interface is more dominant rather than the transport–recombination process inside the mesoporous. The semicircle related to the hole transport layer (PTAA) was not observed in IMVS perhaps due to the limitation of the electrical modulation response of the LED used in the present work.

The appearance of a small semicircle in the low-frequency region (mHz to Hz) could be associated with the Iodide ion migration. Migration and accumulation of ions near the TiO_2_/perovskite interface have recently been reported by Wang et al*.*, which has negative implications for the perovskite solar cell stability^32^. This small semicircle seems larger in the cell-B rather than in the cell-A because, as seen in the XRD data, there is still more unconverted PbI_2_ and hence also the unconverted MAI which may then become the source of free Iodide either inside the formed perovskite nanocrystals or at their grain surface. However, again for a similar reason, because of the different time ranges of the measurements, it is difficult to further analyze and correlate this low semicircle with TPV results and its electronic processes.

**Figure 9:**
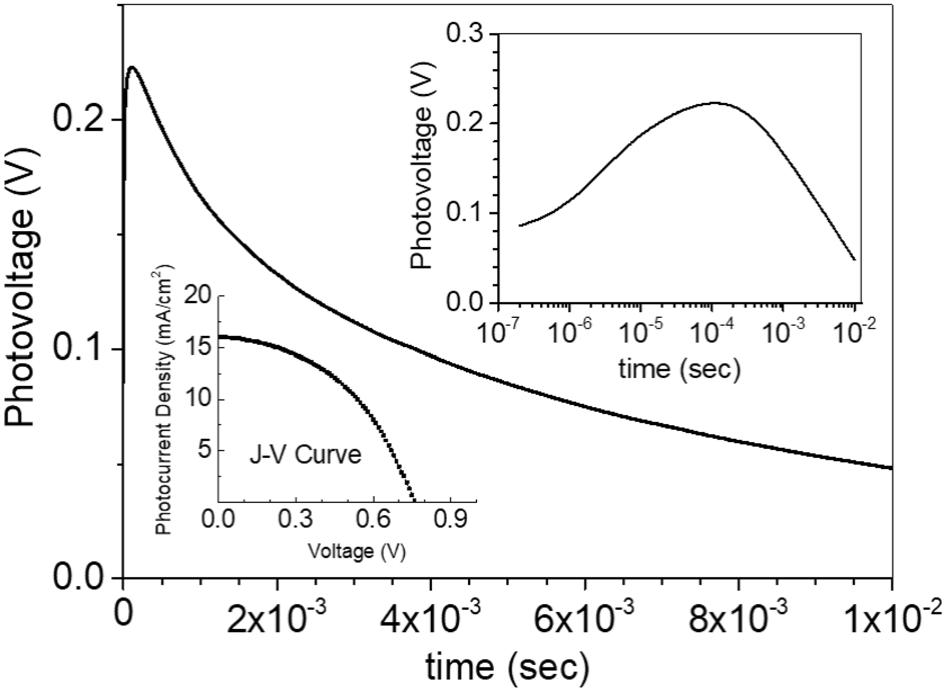
TPV of a cell that was fabricated with the same material and structure as the cell-B. The inset figures show the TPV in the logarithmic scale, showing a slow rising part, and the J–V curve, showing a poor solar cell performance.

The presented data above is the best cell from several batches of fabrication and measurements. As commonly known, the reproducibility and the lifetime of these perovskite solar cells are still low. Figure 9 shows the TPV and J–V curve from a sample prepared with the same structure as the cell-B but different fabrication batch. It is then clear that the aforementioned decay behavior, which is indicated by the slow rising part of TPV decay, can be also observed in the sample with the same material and structure as the cell-B.

In summary, the present experimental results have revealed the relationship between TPV decay characteristics and PSC performance, which were affected by the mesoscopic structure. A detailed examination shows that the TPV decay is composed of a rising part and a decaying part. The rising part can be associated with electron transport inside the perovskite layer, while the decaying part can be associated with the recombination of the accumulated electrons around the perovskite/TiO_2_ interface. A prolonged rising part of TPV decay may correlate with poor PSC performance, where it can be interpreted as electron trapping-detrapping by bulk defects and grain surface states. As consequence, it causes a longer transport time and more electron loss to reach the TiO_2_ layer. On the other hand, the longer decay part can be also correlated with poor PSC performance, which may represent slow back-transfer recombination processes. Such a situation may occur as the result of the presence of a large number of defect traps (including deep-level trap states) at the perovskite/TiO_2_ interface. The TPV data, however, indicates that the rising part plays a more important role that can reflect the solar cell performance. The present results also show that the PSC performance is also greatly affected by charge transport and interfacial processes in a time range longer than the time range of primary processes of photoexcitation.

## Methods

The TiO_2_ compact blocking layer (c-TiO_2_) was prepared using diluted titanium diisopropoxide in isopropanol (with a volume ratio of 1:30). This solution was deposited on a pre-cleaned FTO substrate by spin-coating at 3000 rpm for 30 s. After the spin coating process, the c-TiO_2_ layers were annealed sequentially at 100 °C for 15 min, 300 °C for 15 min, and 500 °C for 30 min. For the mesoporous TiO_2_ layer (mp-TiO_2_), the TiO_2_ paste solution was spin-coated at 5000 rpm for 30 s on FTO/c-TiO_2_. Finally, the TiO_2_ layers were annealed sequentially at 100 °C for 15 min, 300 °C for 15 min, and 500 °C for 30 min. Two types of mp-TiO_2_ layers were prepared, namely: (1) mp-TiO_2_#A that used TiO_2_ pastes diluted with ethanol at a 1:2 vol%. and (2) mp-TiO_2_#B that used TiO_2_ paste diluted with ethanol at 1:16 wt%.

The PSC samples were fabricated by a common technique based on OSPD followed by FDC treatment (the OSPD + FDC technique)^43^. To prepare the perovskite precursor solution, MAPbI_3_ was dissolved in DMF:DMSO (volume ratio = 4:1). The MAPbI_3_ precursor solution was spin-coated onto FTO/c-TiO_2_/mp-TiO_2_ layers at 5000 rpm for 30 s. Finally, the film was annealed at 125 °C for 30 min. The PTAA was then spin-coated as the hole transport layer (HTL) at 3000 rpm for 30 s. The Au electrode of each cell was deposited on top of the HTL by physical thermal evaporation.

**Figure 10:**
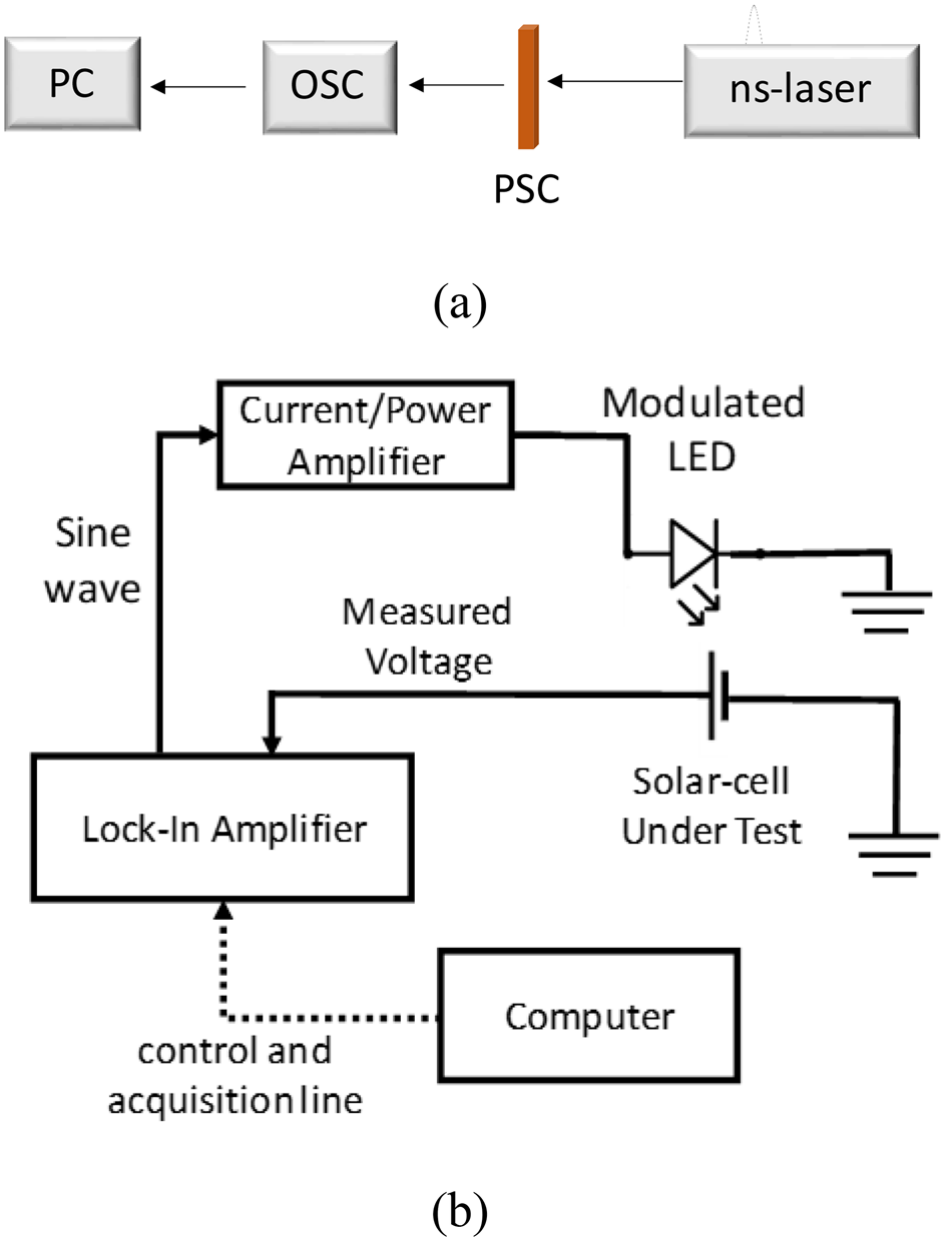
(**a**) A schematic diagram of the TPV measurement system (*PC* personal computer, *OSC* high-definition digital oscilloscope, *PSC* perovskite solar cell, and *ns-laser* nanosecond pulse laser). (**b**) Schematic diagram of the experimental setup for IMVS measurements.

The photovoltaic characteristics of the solar cell were measured by a standard solar simulator with a light source intensity of 100 mW/cm^2^ (1 Sun, 1.5 AM). The measurements of transient photovoltage (TPV) were measured using the setup shown in Fig. 10a. A Q-switched Nd-YAG Nd/YAG laser (CryLas Gmbh FDSS 532–150) operating at a wavelength of 532 nm, pulse duration of ~ 1 ns, and maximum output energy 150 µJ has been used as the pulse laser source. The generated transient voltage was recorded using a high-definition digital oscilloscope (Teledyne LeCroy HDO4054), with the maximum sample rate 2.5 GS/s per single-shot and the resistance and capacitance input of 1 MΩ and 15 pF, respectively.

The IMVS measurements were conducted by using a modulated LED and a lock-in amplifier (Stanford Research 850) with the experimental setup shown diagrammatically in Fig. 10b A current/power amplifier (Rigol PA1011 10 W) was used to amplify sine wave generated from the lock-in up to hundreds mA such that sufficiently enough to supply the LED. The frequency range of LED modulation was 0.1 Hz–100 KHz. The control and data acquisition were performed by a personal computer.

## Supplementary information

Supplementary Information.
