# Supplementary material for: Revealing the charge carrier kinetics in perovskite solar cells affected by mesoscopic structures and defect states from simple transient photovoltage measurements
Source: Sci Rep. 2020 Nov 5;10:19197. doi: 10.1038/s41598-020-74603-x (PMC7645725; doi:10.1038/s41598-020-74603-x)
Supplement: Supplementary file 1 — Supplementary Information. [file 41598_2020_74603_MOESM1_ESM.docx]

**REVEALING THE CHARGE CARRIER KINETICS IN PEROVSKITE SOLAR CELLS AFFECTED BY MESOSCOPIC STRUCTURES AND DEFECT STATES FROM SIMPLE TRANSIENT PHOTOVOLTAGE MEASUREMENTS**

Rahmat Hidayat*^,1^, Adhita Asma Nurunnizar^1^, Alvin Fariz^1^, Herman^1^, Erlyta Septa Rosa^2^, Shobih^2^, Tomohisa Oizumi^3^, Akihiko Fujii^3^, Masanori Ozaki^3^

^1^ Institut Teknologi Bandung, Physics of Magnetism and Photonics Research Division, Physics Program Study, Faculty of Mathematics and Natural Sciences, Jl. Ganesha 10, Bandung 40132, West Java, Indonesia

^2^ Indonesian Institute of Sciences, Research Center for Electronics and Telecommunication, Jl. Sangkuriang Komp. LIPI Gd.20 Bandung 40135, West Java, Indonesia

^3^ Osaka University, Department of Electrical, Electronics, and Information Engineering, Faculty of Engineering, Yamada-Oka 2-1, Suita, Osaka 565-0871, Japan

* corresponding author: [rahmat@fi.itb.ac.id](mailto:rahmat@fi.itb.ac.id)

**Supplementary Information**

Figure S1. The optical absorption (UV-Vis) spectra of MAPbI_3_ perovskite spin-coated onto the mp-TiO_2_#A and mp-TiO_2_#B layers.


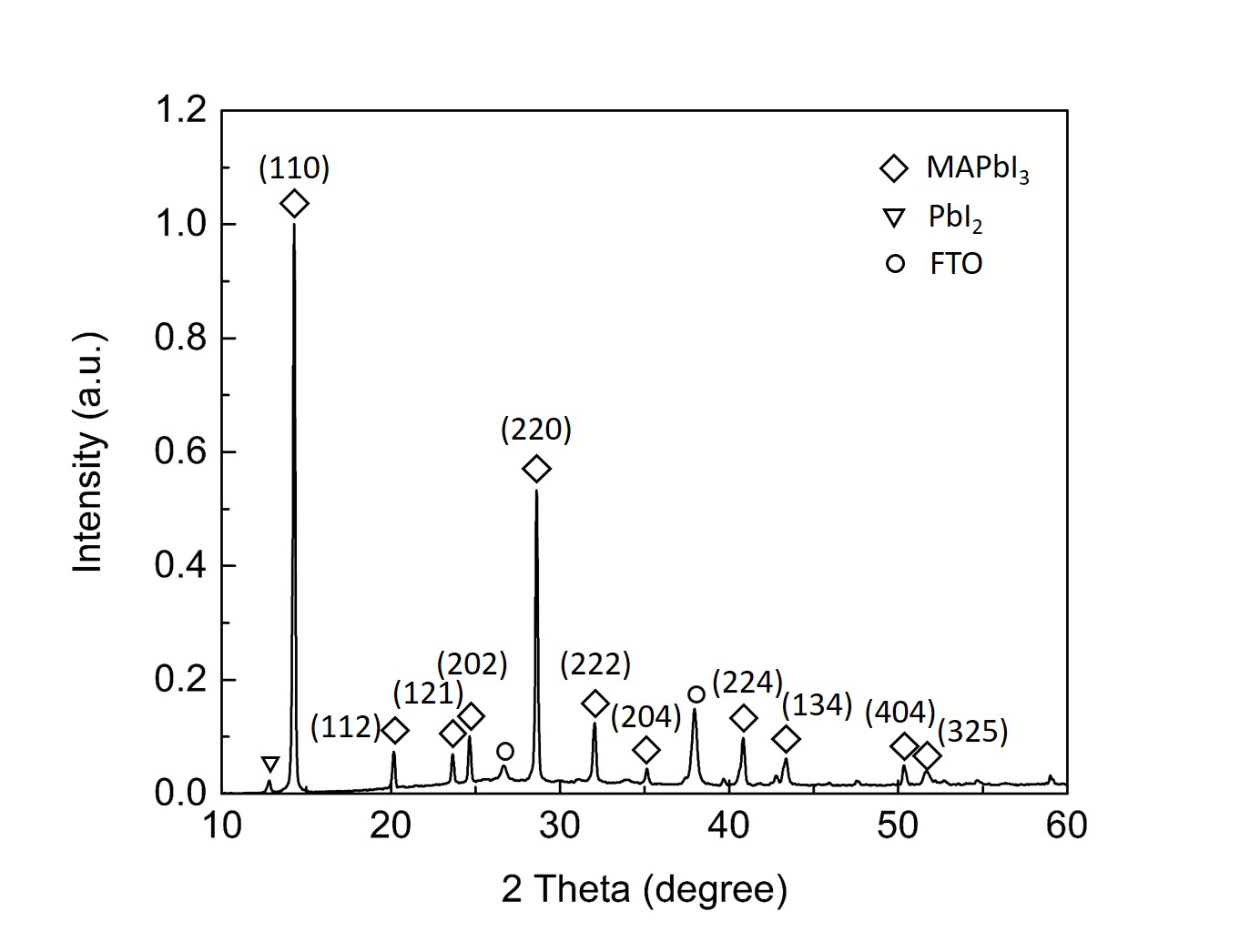


(a) (b)

Figure S2. The XRD pattern of (a) MAPbI_3_ perovskite on the mp-TiO_2_#A and (b) MAPbI_3_ perovskite on the mp-TiO_2_#B, showing almost identical tetragonal perovskite structure formation in those samples. *Inset*: the (110) peak shown on the expanded scale for showing the width difference of those peaks.

Figure S3. The external quantum efficiency (EQE) of the cell-B. The absorption spectrum of the similar perovskite layer on mp-TiO_2_#B was also shown for comparison of its light absorption range.

**Methods**

The measurement of optical (UV-Vis) absorption was conducted by using a CCD based UV-Vis spectrophotometer (Ocean Optics, USB 2000) and a tungsten lamp as its light source. The XRD measurements were done by using the Bruker D8 Advance diffractometer, which uses Cu Kα monochromatic X-ray source (λ = 0.15418 nm). The external quantum efficiency (EQE) measurements were carried out by a photosensitivity measurement system (Bunkoukeiki, SM-250NA), which was calibrated using a standard cell.
